# Editorial: Decipherment of genetic architecture of psychiatric disorders via combination of multi-omics data

**DOI:** 10.3389/fgene.2024.1430517

**Published:** 2024-07-09

**Authors:** Jian-Wei Jiang, Zhong-Cheng Jian, Mary Miu Yee Waye, Wei-Cheng Liang, Miao-Xin Li, Xiao-Pei Shen, Shi-Tao Rao

**Affiliations:** ^1^ Department of Bioinformatics, Fujian Key Laboratory of Medical Bioinformatics, Institute of Precision Medicine, School of Medical Technology and Engineering, Fujian Medical University, Fuzhou, China; ^2^ Department of Pediatrics, Longyan People Hospital of Fujian, Longyan, China; ^3^ Croucher Laboratory for Human Genomics, The Nethersole School of Nursing, The Chinese University of Hong Kong, Shatin, Hong Kong SAR, China; ^4^ Vaccine Research Institute, The Third Affiliated Hospital of Sun Yat-sen University, Sun Yat-sen University, Guangzhou, China; ^5^ Zhongshan School of Medicine, Center for Precision Medicine, Sun Yat-sen University, Guangzhou, China; ^6^ Key Laboratory of Tropical Disease Control (SYSU), Ministry of Education, Guangzhou, China; ^7^ School of Biomedical Sciences, The Chinese University of Hong Kong, Shatin, Hong Kong SAR, China

**Keywords:** mental illness, genetic epidemiology, genetic factors, psychological traits, correlated risk diseases

Mental illness is a health condition characterized by changes in thinking, emotion, or behavior that result in distress or impairments in social, occupational, or familial functioning. In the United States, more than one in five adults experienced at least one form of mental illnesses in 2021 (National Institute of Mental Health, 2023). Over the past three decades, the number of disability-adjusted life years (DALYs) associated with mental illnesses has risen from 43.9 million to 69.0 million (Chen et al., 2024). In 2019, mental disorders were ranked as the eighth most significant disease burden in Asia. Among various mental illnesses, major depressive disorder (MDD) accounts for a significant proportion (37.2%) of the age-standardized DALY rates in Asia, followed by anxiety disorder (21.5%). Schizophrenia, a frequently debilitating mental illness, affects individuals worldwide, with a lifetime risk of approximately 1% (Lam et al., 2019). Recent estimates indicate that the number of diagnoses of Alzheimer’s disease and related dementias among adults aged 65 years and older in the United States will rise to 13.9 million in 2060, compared to 5.0 million in 2014 (Matthews et al., 2019). Given these circumstances, it is imperative that we conduct in-depth investigations into the risk factors and pathogenesis of mental illnesses to mitigate their devastating effects.

**FIGURE 1:**
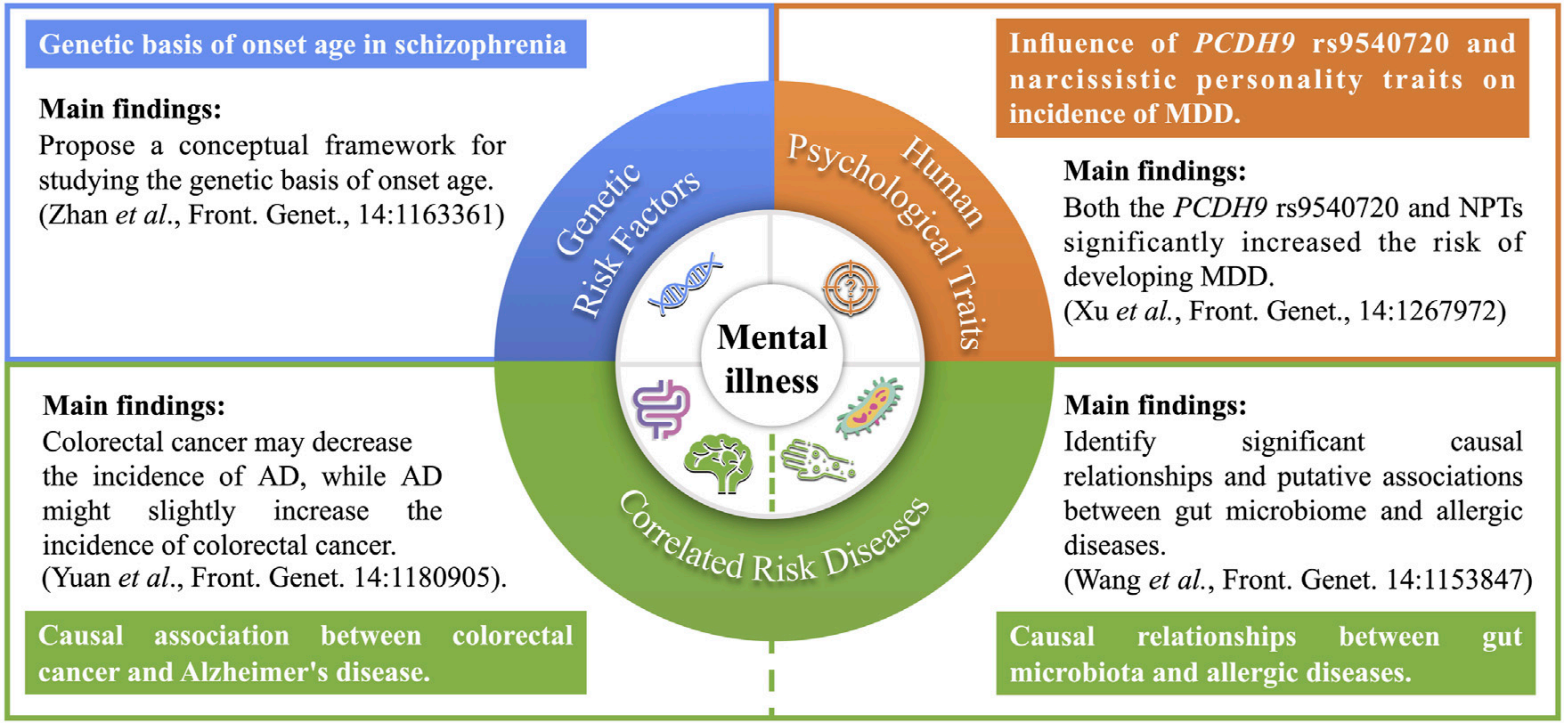
Summary of main contents covered in the four published articles within the Research Topic. AD: Alzheimer’s disease; *PCDH9*: protocadherin 9; NPTs: narcissistic personality traits; MDD: major depressive disorder.

It is widely recognized that mental illnesses are influenced by a combination of genetic variants, environmental factors, and psychological correlates (Arango et al., 2018). Although mental illnesses typically do not follow the standard patterns of Mendelian inheritance, genetics and genetic epidemiology have proven invaluable in unraveling their complex mechanisms and identifying the risk factors. Additionally, common human diseases, such as allergic diseases and various cancers, have been identified as significant factors in the development of mental illnesses (Budu-Aggrey et al., 2021). To identify risk factors and gain a deeper understanding of the pathophysiological mechanisms underlying multiple mental illnesses, this Research Topic comprises four articles (Figure 1).

Zhan et al. focused their research on unraveling the genetic basis of age at onset (AAO) in schizophrenia, recognizing the multiple theoretical and clinical implications associated with this Research Topic. Understanding the genetic underpinnings of AAO in schizophrenia can shed light on the biological mechanisms of the disorder, facilitate prediction of schizophrenia onset, and aid in the subtyping of schizophrenia. Through a meticulous review of the existing evidence on the genetic basis of AAO, the authors not only discussed the potential significance of AAO as an important phenotypic dimension of the disorder, but also provided an overview of the methodologies, sample characteristics, findings, and limitations of previous genetic research on AAO. Furthermore, they proposed a conceptual framework to investigate the genetic basis of AAO, highlighting its potential clinical implications for future genetic research endeavors.

Xu et al. conducted a study with the objective of examining the influence of a specific polymorphism, rs9540720, in the protocadherin 9 (*PCDH9*) gene, as well as narcissistic personality traits (NPTs), on the incidence of MDD in Chinese first-year university students. Additionally, they explored the interactive effects of this polymorphism and NPTs on MDD incidence. Utilizing univariate logistic regression analyses, they discovered that both the *PCDH9* rs9540720 polymorphism and NPTs significantly increased the risk of developing MDD. However, the subsequent interaction analyses did not reveal any significant multiplicative or additive interaction effects between the polymorphism and NPTs. This research highlights the importance of studying the impact of genetic and psychological factors on MDD incidence in college students, as it holds significant social value and can potentially contribute to the prevention of MDD in this specific population.

Yuan et al. aimed to elucidate the causal correlations between colorectal cancer and Alzheimer’s disease (AD) using genetic summary data from large-scale genome-wide association studies (GWASs). The authors obtained three GWAS datasets comprising individuals of European ancestry, with sample sizes exceeding 400,000 subjects. With the widely recognized Mendelian Randomization (MR) method, the authors revealed that colorectal cancer may decrease the incidence of AD, while AD might slightly increase the incidence of colorectal cancer. Furthermore, sensitivity and heterogeneity analyses confirmed the reliability of these findings. Considering that both colorectal cancer and AD are common life-threatening diseases affecting the elderly population, these findings underscore the importance of early diagnosis and treatment for both patient groups.

Wang et al. conducted a groundbreaking study that investigated the interplay between host genetics, gut microbiome composition, and allergic diseases, while also estimating the potential causal relationships among these factors. Utilizing the MR IVW method, the authors successfully identified several significant causal relationships and putative associations between the gut microbiome and allergic diseases. The gut microbiome is increasingly recognized as an important risk factor for allergic diseases, as it influences the host’s immune response and interacts with host genetic factors. The findings of this study shed light on the comprehensive interactions between the host and the gut microbiome in individuals with allergies.

The four studies highlighted in this Research Topic provide valuable insights into the genetic basis of mental illness and the identification of novel risk factors. However, there are several areas where these studies could be improved to enhance their findings. Firstly, in the review on the genetic basis of AAO in schizophrenia, a quantitative assessment of the retrieved data could be conducted to provide more robust results. Additionally, addressing the issue of publication bias would strengthen the overall conclusions. Secondly, the study investigating the role of the interaction between genetic factors and psychological traits on the incidence of MDD relied primarily on questionnaire data, which may introduce recall bias and inaccurate diagnosis (Cai et al., 2020). Utilizing more objective and reliable methods of data Research Topic would improve the accuracy of the findings. Furthermore, the use of MR methods in some studies may be limited by small sample sizes and genetic background heterogeneity, potentially leading to inconsistent results between studies. Increasing sample sizes and considering population-specific genetic factors would enhance the reliability and generalizability of the MR findings. To further advance our understanding of the genetic basis and risk factors of mental illnesses, it would be beneficial to address these Research Topic and engage more researchers in the field. Additionally, the integration of multi-omics data at various levels, including genomics, transcriptomics, proteomics, and metabolomics, could provide innovative insights into early diagnosis, personalized medicine, and the identification of druggable genetic targets for mental illness (Li et al., 2022).
